# Using Unmanned Aerial Vehicle-Based Multispectral Image Data to Monitor the Growth of Intercropping Crops in Tea Plantation

**DOI:** 10.3389/fpls.2022.820585

**Published:** 2022-02-25

**Authors:** Yujie Shi, Yuan Gao, Yu Wang, Danni Luo, Sizhou Chen, Zhaotang Ding, Kai Fan

**Affiliations:** ^1^Tea Research Institute, Qingdao Agricultural University, Qingdao, China; ^2^Jinan Agricultural Technology Promotion Service Center, Jinan, China

**Keywords:** UAV, multispectral, machine learning, leaf area index, above-ground biomass

## Abstract

Aboveground biomass (AGB) and leaf area index (LAI) are important indicators to measure crop growth and development. Rapid estimation of AGB and LAI is of great significance for monitoring crop growth and agricultural site-specific management decision-making. As a fast and non-destructive detection method, unmanned aerial vehicle (UAV)-based imaging technologies provide a new way for crop growth monitoring. This study is aimed at exploring the feasibility of estimating AGB and LAI of mung bean and red bean in tea plantations by using UAV multispectral image data. The spectral parameters with high correlation with growth parameters were selected using correlation analysis. It was found that the red and near-infrared bands were sensitive bands for LAI and AGB. In addition, this study compared the performance of five machine learning methods in estimating AGB and LAI. The results showed that the support vector machine (SVM) and backpropagation neural network (BPNN) models, which can simulate non-linear relationships, had higher accuracy in estimating AGB and LAI compared with simple linear regression (LR), stepwise multiple linear regression (SMLR), and partial least-squares regression (PLSR) models. Moreover, the SVM models were better than other models in terms of fitting, consistency, and estimation accuracy, which provides higher performance for AGB (red bean: *R*^2^ = 0.811, root-mean-square error (RMSE) = 0.137 kg/m^2^, normalized RMSE (NRMSE) = 0.134; mung bean: *R*^2^ = 0.751, RMSE = 0.078 kg/m^2^, NRMSE = 0.100) and LAI (red bean: *R*^2^ = 0.649, RMSE = 0.36, NRMSE = 0.123; mung bean: *R*^2^ = 0.706, RMSE = 0.225, NRMSE = 0.081) estimation. Therefore, the crop growth parameters can be estimated quickly and accurately using the models established by combining the crop spectral information obtained by the UAV multispectral system using the SVM method. The results of this study provide valuable practical guidelines for site-specific tea plantations and the improvement of their ecological and environmental benefits.

## Introduction

Intercropping, as the essence of traditional agriculture, has the advantages of increasing yield and quality (Mao et al., 2014; Egesa et al., 2016), promoting the utilization of nutrient resources (Rivest et al., 2010; Crème et al., 2016; Davies et al., 2016), increasing biodiversity (Bainard et al., 2011; Sanaa et al., 2016), and reducing pests and weeds (Brooker et al., 2015; Lopes et al., 2016). Tea plants [*Camellia sinensis* (L.) O. Kuntze] are cultivated worldwide as an economical woody plant, which grow in warm, humid, and light scattering regions. The different intercropping patterns of tea plantations, such as tea-fruit and tea-soybean intercropping, will be more in line with the biological characteristics of tea plant growth by improving microenvironment and resource utilization. Previous studies have shown that diverse agroforestry-tea intercropping systems, such as tree/tea and soybean/tea cannot only regulate the ecological environment of tea plantation, improve the soil nutrition, but also reduce the occurrence of diseases and insect pests and grass, and achieve high yield and quality (Sedaghathoor and Janatpoor, 2012; Li et al., 2019). However, the intercropping density and the growth status of intercropping crops have a great influence on the growth of tea plants (Natarajan and Willey, 1980; Huang et al., 2019). A better understanding of the growth and development of intercropping crops is of great significance for guiding young tea plantation intercropping techniques and improving planting benefits.

Aboveground biomass (AGB) and leaf area index (LAI) are two main parameters of crop growth, which can reflect the growth status of legumes intercropped in young tea plantations, thus contributing to production management in tea plantations (Li et al., 2015; Liu B. et al., 2017). Rapid and accurate estimation of these two parameters can provide a strong basis for the timely formulation of management measures for young tea plantations (Li B. et al., 2020). However, the traditional crop growth assessment method is based on destructive sampling, which is to manually collect data samples in the field, or use field measuring instruments to evaluate crops (Freeman et al., 2007; Yue et al., 2018; Afrasiabian et al., 2020). Although this method is accurate, it is destructive, labor-intensive, time-consuming, and not operationally feasible for large-scale spatial and temporal measurements (Wang et al., 2017). Another relatively new method is to use instruments for measurement, which is less destructive to crops, but external factors have a certain impact on experimental equipment, and it is also difficult to apply to rapid monitoring of field crops.

In recent years, high-throughput non-destructive plant phenotyping techniques based on UAV are becoming a powerful tool for crop monitoring, due to the advantages of convenient operation, high spatial and temporal resolution, and reasonable spatial coverage, such as crop plot detection (Liu H. et al., 2017), crop growth status monitoring (Pölönen et al., 2013; Harkel et al., 2019; Maimaitijiang et al., 2019), crop yield prediction (Zhou et al., 2017; Gilliot et al., 2020; Li B. et al., 2020), and plant water status assessment (Romero et al., 2018). Machine learning, as an important data analysis method, has been used to establish crop remote sensing estimation models combined with spectral parameters of remote sensing images. For example, Jin et al. (2015) used a vegetation index (VI) and radar parameter to accurately estimate the LAI (*R*^2^ = 0.83) and biomass (*R*^2^ = 0.90) of winter wheat using partial least-squares regression (PLSR). Devia et al. (2019) used an unmanned aerial vehicle (UAV)-based multispectral system for aerial crop monitoring to combine seven VIs of rice growth in a multivariate regression model to estimate rice biomass. Furthermore, it was confirmed that this method could estimate crop biomass in a large area with an average correlation coefficient of 0.76. Han et al. (2019) pointed out that the random forest (RF) model derived from the crop surface model using VIs and crop height correlation indicators can predict corn biomass (*R*^2^ = 0.699, root-mean-square error (RMSE) = 1.2), and its accuracy is slightly higher than that of the backpropagation artificial neural network (ANN) and stepwise multiple linear regression (SMLR) models. Qi et al. (2020) developed a model for the estimation of peanut LAI by using a backpropagation neural network (BPNN) with UAV-based multispectral image data (*R*^2^ = 0.968, RMSE = 0.165). Tatsumi et al. (2021) constructed a tomato biomass estimation model using red-green-blue (RGB) and multispectral image data acquired from UAV with feature variable selection and machine learning and improved the estimation accuracy (rRMSE = 8.8–28.1%). Similarly, Jiang et al. (2019) established a model for the estimation of rice biomass by using RGB and multispectral image data obtained from UAV and further improved the estimation accuracy of the model by combining meteorological data with RF (*R*^2^ = 0.92, RMSE = 126.28 g/m^2^).

However, there were few reports on the use of UAV-based multispectral image data combined with machine learning to monitor crop growth of tea plantations, and it is difficult to provide valuable data support and practical guidance for site-specific management decisions and the construction of smart tea plantations. Therefore, this study attempts to use UAV-based multispectral imagery combined with ground-measured sample data to explore the feasibility of estimating AGB and LAI using the spectral parameters in intercropping tea plantations. The spectral parameters sensitive to crop growth response were selected according to the correlation analysis. Then, the remote sensing monitoring models of intercropping crop growth parameters suitable for young tea plantation were constructed using machine learning, and the estimation performance of five machine learning models was evaluated: (1) Simple linear regression (LR), (2) SMLR, (3) PLSR models, (4) support vector machine (SVM), and (5) BPNN. We hypothesized that the SVM method can simulate both linear and non-linear relationships between multiple independent variables and one factor. Compared with other modeling methods, the SVM model should have a higher degree of explanation for AGB and LAI. It is hoped that the results of this study can provide basic data and theoretical support for the growth monitoring of crops in young tea plantations in order to provide valuable practical guidelines for site-specific tea plantations and the improvement of their ecological and environmental benefits.

## Materials and Methods

### Study Area and Experimental Design

**FIGURE 1:**
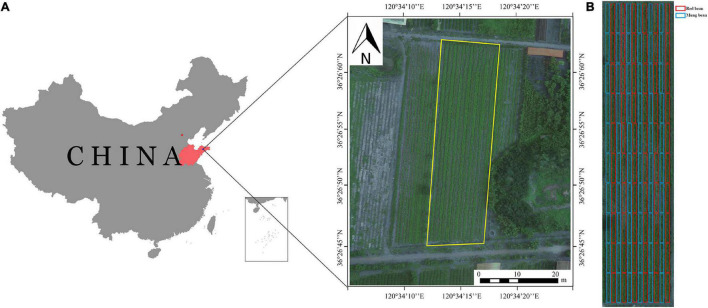
Research area. **(A)** The location of the experiment area; **(B)** experimental design.

The field experiment was conducted at the tea research demonstration base of Qingdao Agricultural University (36°26 N, 120°34 E, average altitude 54.47 m a.s.l.). The area has a warm temperate continental monsoon climate, with precipitation mostly occurring during summer and autumn and a large temperature difference between day and night. The average annual temperature is 12.1° (the annual maximum/minimum temperature is 38.6/−18.6°), and the annual average precipitation is 708.9 mm. The experimental tea plantation covers an area of 100 m × 30 m, with a soil pH of 6.5. The location diagram of the experiment area is shown in Figure 1A.

The tea plants planted in the tea plantation are half-year seedlings and the variety was Zhongcha 108, with a total of 11 rows. In early June 2020, mung bean and red bean were planted in the tea plantation, and the varieties, namely, Zhonglv 4 and Qidonghong were used. Red bean (*Vigna angularis* L.) and mung bean (*V. radiata* (L.) Wilczek) were planted in rotation with 6 rows each. Each row was divided into 10 plots, with a total of 120 plots. The specific test design is shown in Figure 1B.

### Unmanned Aerial Vehicle Imagery Data Acquisition and Preprocessing

Multispectral cameras and accessories were mounted on a UAV platform (DJI M200 V2, DJI, Shenzhen, China) during data collection. The UAV has four propellers, is equipped with two 7,660 mAh (22.8 V) batteries with a battery life of 38 min, and can maintain stability at low speed and low altitude; for the data acquisition, the takeoff mass was 5.5 kg. Images were taken at 25 m above ground level (AGL) at a speed of 1.5 m/s. The collection dates were July 24 and August 11, 2020.

Multispectral images were acquired using a multispectral camera MS600 (Yusense, Qindao, Shandong, China), which has a resolution (effective pixels) of 1,280 × 960 pixels. The multispectral camera used in this experiment was equipped with six spectral wavebands, namely blue, green, red, red edge, and two near-infrared wavebands (Table 1). A downward light sensor system was installed horizontally on the top of the UAV to measure the environmental irradiance and the readings of post-calibrate reflectance. As another source of radiometric calibration data, the standard panel attached to the multispectral camera was used for image calibration on the ground before each flight. Images in this study were captured in sub-centimeter pixel resolution, and the flight survey was configured with an 80% side and 80% forward overlap. The original multispectral images obtained from each aerial photography operation were processed using Yusense map V1.0 software (Yusense, Qindao, Shandong, China) to generate a complete multispectral image. Then, the average digital number (DN) values of the six bands of each experimental cell are extracted using ENVI 5.2 software (Research Systems Inc., Boulder Co., United States) for subsequent processing.

**TABLE 1:** Center wavelength and full width at half maximum (FWHM) bandwidth of each spectral band of the multispectral camera.

Spectral band	Color	Sample	Center wavelength (nm)	Bandwidth FWHM (nm)
Blue	Blue	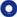	450	25
Green	Green	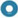	555	25
Red	Red	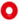	660	25
Red edge	Pink	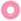	710	25
Near infrared	Light purple	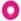	840	25
Near infrared	Purple	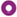	940	25

### Ground Data Acquisition

**FIGURE 2:**
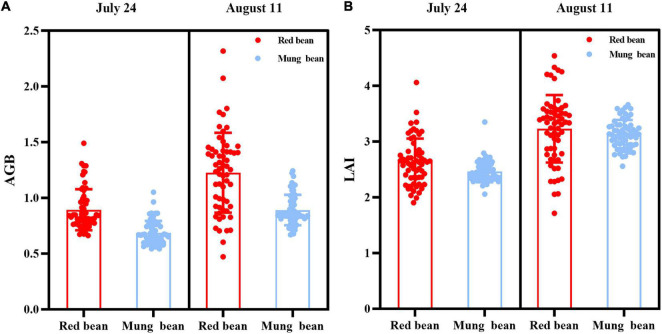
The ground-truth data for leaf area index (LAI) and aboveground biomass (AGB) of intercropping crops. **(A)** AGB of red bean and mung bean; **(B)** LAI of red bean and mung bean.

Field measurements were conducted on the same days as the UAV surveys to provide ground-truth data. To measure the LAI of red bean and mung bean accurately, a place with uniform crop growth (1 m × 1 m) in each plot was selected to measure the LAI using CI-110 plant canopy digital image analyzer (CID Bio-Science Inc., WA, United States). When measuring LAI, direct sunlight was avoided. First, a blank value was measured above the crop canopy, and then four values were randomly measured below the crop canopy. The average LAI of mung bean and red bean in the community was obtained by maintaining the lens level throughout the measurement, and the results are shown in Figure 2.

After the measurement of LAI, mung bean or red bean were randomly selected from experimental plots, which were intercepted from the height of 1 cm above the ground, and the total number of plants in the sampling area was measured. The sample was placed in a paper bag, and the fresh biomass of the sample was measured immediately. The paper bag was placed in an oven at 80° for 24 h and maintained in a constant mass state. Then the sample was weighed to determine the dry mass to estimate the total biomass of the whole plot, and the results are shown in Figure 2.

### Selection of Spectral Parameters

A spectral parameter should combine the reflectance of different bands with a VI in a certain way, which can reduce the influence of background environmental information on the crop canopy spectrum. According to previous studies, we selected 22 VIs and combined them with the 6 spectral bands of the MS600 multispectral camera to estimate the AGB and LAI of red bean and mung bean. Specific spectral parameters are shown in Table 2.

**TABLE 2:** The spectral parameters used in this study.

Spectral parameters	Calculation formula	References
B.450	/	/
G.555	/	/
R.660	/	/
RE.710	/	/
NIR.840	/	/
NIR.940	/	/
DVI	NIR.840-G.555	Naito et al., 2017
NDVI	(NIR.840-R.660)/(NIR.840+R.660)	Rouse et al., 1974
EVI	2.5*(NIR.940-G.555)/(NIR.940+6*R.660-7.5B.450+1)	Prabhakara et al., 2015
GNDVI	(NIR.940-G.555)/(NIR.940+G.555)	Wang et al., 2007
PPR	(G.555-B.450)/(G.555+B.450)	Metternicht, 2003
SIPI	(NIR.940-B.450)/(NIR.940-R.660)	Penuelas et al., 1995
RECI	NIR.840/RE.710-1	Kanke et al., 2016
Red edge NDVI	(NIR.940-RE.710)/(NIR.940+RE.710)	Kanke et al., 2016
MERIS Terrestrial Chlorophyll Index (MTCI)	(NIR.840-RE.710)/(RE.710-R.660)	Panigada et al., 2010
Modified chlorophyll absorption ratio index (MCARI)	[RE.710-R.660-0.2(RE.710-R.660)] *(RE.710/R.660)	Wu et al., 2008
Triangular vegetation index (TVI)	0.5*[120*(NIR.840-G.555)-200*(R.660-G.555)]	Haboudane et al., 2004
Modified triangular vegetation index (MTVI2)	1.5*[1.2*(NIR.840-G.555)-2.5*(R.660-G.555)]/[(12*NIR.880+1)^2^-[6*NIR.880-5*(R.660)^2^]-0.5]^1/2^	Haboudane et al., 2004
Transformed chlorophyll absorption reflectance index (TCARI)	3*[(RE710-R.660)-0.2*(RE.710-G.555) *(RE.710/G.555)]	Haboudane et al., 2004
Optimization of soil-adjusted vegetation index (OSAVI)	1.16*(NIR.840-R.660)/(NIR.840+R.660+0.16)	Rondeaux et al., 1996
Ratio vegetation index (RVI1)	NIR.840/R.660	Kanke et al., 2016
PPR/NDVI	PPR/NDVI	Jin et al., 2017
SIPI/RVI1	SIPI/RVI1	Jin et al., 2017
Modified non-linear vegetation index (MNLI)	1.5*[(NIR.840)^2^-R.660)]/(NIR.842)^2^+R.660+0.5	Yang Z. et al., 2008
Soil-adjusted vegetation index (SAVI)	(NIR.840-R.660)/(NIR.840+R.660+0.5)	Pinty and Verstraete, 1992
Modified simple ratio (MSR)	(NIR.840/R.660-1)/[(NIR.840/R.660)^1/2^+1]	Wu et al., 2008
Non-linear vegetation index (NLI)	[(NIR.840)^2^-R.660]/[(NIR.840)^2^+R.660]	Goel and Qin, 1994
Renormalized difference vegetation index (RDVI)	(NIR.840-R.660)/(NIR.840+R.660)^1/2^	Tucker, 1979

### Data Analysis

In this study, 120 datasets of red bean and mung bean were collected. Each dataset was composed of ground measurement data and UAV remote sensing data. In data analysis, three-fourth (90 datasets) and one-fourth (30 datasets) of the total data were divided into training sets and test sets, respectively. In the training sets, the LR method was used to establish growth parameter estimation models based on a single spectral parameter, and the SMLR method was used to establish growth parameter estimation models based on multiple spectral parameters. These two different established models were evaluated using the test datasets. The feasibility of the models was evaluated by the coefficient of determination (*R*^2^), root-mean-square error (RMSE), and normalized RMSE (NRMSE). A larger *R*^2^ value indicates a better model fit, while smaller RMSE and NRMSE values indicate a higher model accuracy. Finally, the estimation models of AGB and LAI were established by using three machine learning methods: PLSR, SVM, and BPNN. In the process of model building, the random 10-fold cross-validation method was used to divide 120 sample data into 10 parts. Each time, 90% of all samples was used to fit the model, and the remaining 10% was used as a test set to estimate performance metrics. This process was repeated ten times, and each model was run 100 times in total. The mean values of *R*^2^, RMSE, and NRMSE were calculated to evaluate the accuracy of AGB and LAI estimation models. The values of *R*^2^, RMSE, and NRMSE were calculated using the following formulas (1)–(3), respectively:


(1) $$R^{2}=1-\frac{\sum_{i=1}^{n}{\left(x_{i}-y_{i}\right)}^{2}}{\sum_{i=1}^{n}{\left(x_{i}-\bar{x}\right)}^{2}}$$

(2) $$R\,M\,S\,E=\sqrt{\frac{\sum_{i=1}^{n}{\left(y_{i}-x_{i}\right)}^{2}}{n}}$$

(3) $$N\,R\,M\,S\,E=\frac{R\,M\,S\,E}{\bar{X}}$$

where *x*_*i*_ is the measured AGB or LAI for red bean and mung bean, $\bar{\text{x}}$ is the average measured AGB or LAI, *y*_*i*_ is the AGB or LAI predicted by the model, and *n* is the number of data points.

## Results

### Correlation Analysis Between Spectral Parameters With Growth Parameters

**FIGURE 3:**
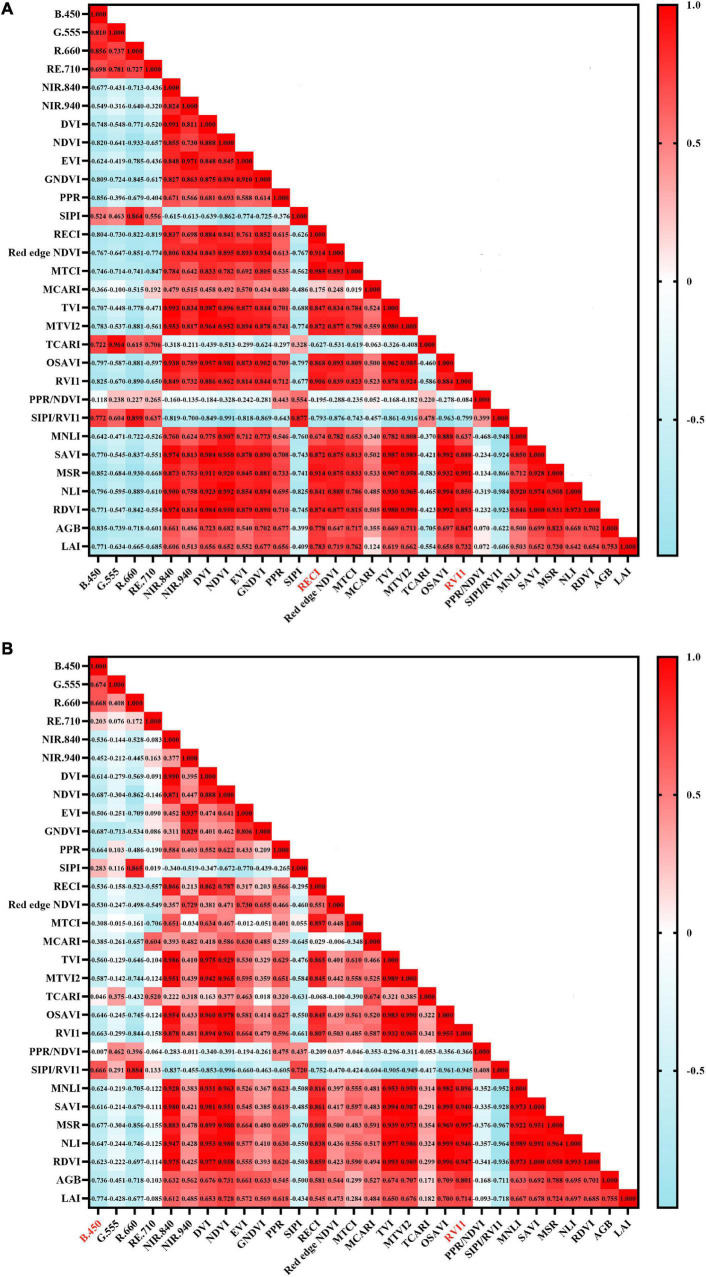
Correlation coefficients between spectral parameters and growth parameters (AGB and LAI) of intercropped crops. **(A)** AGB and LAI of red bean; **(B)** AGB and LAI of mung bean.

To select the spectral parameters that are highly correlated with the growth parameters (AGB and LAI) of red bean and mung bean, the correlation analysis between 28 spectral parameters and the growth parameters of red bean and mung bean (Figure 3) was carried out. For the AGB and LAI of red bean, the spectral parameters with the strongest correlation were RVI1 and red-edge chlorophyll index, and their correlation coefficients were 0.847 and 0.783, respectively. For the AGB and LAI of mung bean, the spectral parameters with the strongest correlation were RVI1 and B.450, and their correlation coefficients were 0.801 and 0.774, respectively. In general, most of the spectral parameters selected in this study had a strong correlation with the growth parameters, which can be used for the modeling and inversion of AGB and LAI of red bean and mung bean.

### Estimation of Aboveground Biomass and Leaf Area Index Using Optimal Spectral Parameters Combined With Simple Linear Regression

**FIGURE 4:**
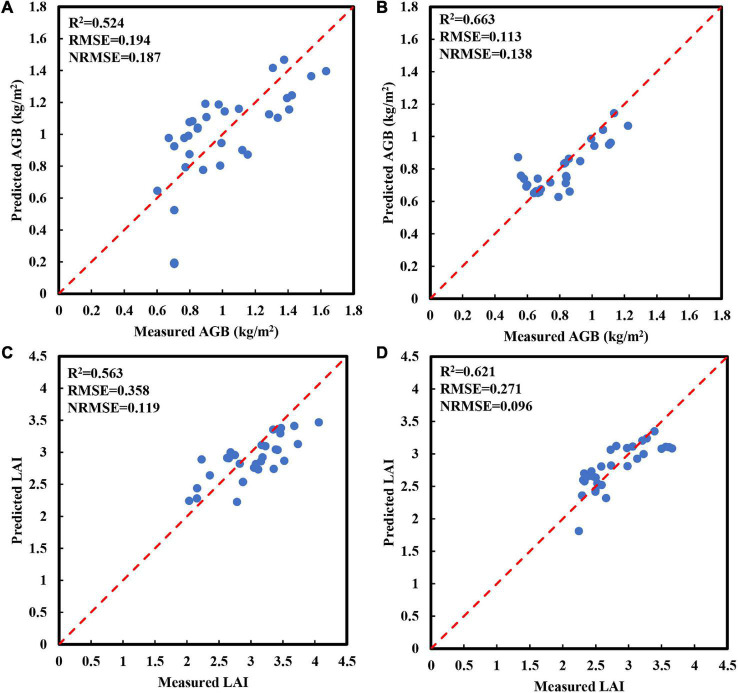
Relationship between the predicted and measured AGB and LAI obtained by using linear regression (LR) methods using the optimal spectral parameters in the test set. **(A)** AGB of red bean; **(B)** AGB of mung bean; **(C)** LAI of red bean; **(D)** LAI of mung bean. The red line is a 1:1 line.

To evaluate the direct relationship between spectral parameters and crop growth parameters, the LR method was used to establish AGB and LAI estimation models of red bean and mung bean in the training set using the optimal spectral parameters screened by correlation analysis (Table 3). Then, we verified the models with a test set (Figure 4). The training results showed that RVI1 could explain 76.1% (RMSE = 0.168 kg/m^2^, NRMSE = 0.157) and 62.6% (RMSE = 0.088 kg/m^2^, NRMSE = 0.113) of AGB variation in red bean and mung bean, respectively. As for LAI, the optimal spectral parameter red-edge chlorophyll index (RECI) could explain 63.4% (RMSE = 0.376, NRMSE = 0.129) of the LAI variation in red bean and B.450 could explain 59.1% (RMSE = 0.25, NRMSE = 0.09) of the LAI variation in mung bean. In addition, for growth parameters of red bean, these models deteriorated with the test dataset and the explanatory degree for AGB and LAI variation decreased to 52.4% (RMSE = 0.194 kg/m^2^, NRMSE = 0.187) and 56.3% (RMSE = 0.357, NRMSE = 0.119), respectively (Figures 4A,C). In contrast, for growth parameters of mung bean, the models performed better with the test dataset and the explanatory degree for AGB and LAI variation increased to 66.3 and 62.1%, respectively. At the same time, the values of RMSE increased to 0.113 and 0.271, and the values of NRMSE increased to 0.138 and 0.096, respectively (Figures 4B,D).

**TABLE 3:** Performance indicators of the AGB and LAI estimation models established by the LR method using the optimal spectral parameters in the training set.

Growth parameters	Intercropping crops	Optimal spectral parameters	Regression equation	Modeling accuracy		
				*R* ^2^	RMSE	NRMSE
AGB (kg/m^2^)	Red bean	RVI1	AGB = 0.059*RVI1+0.313	0.761	0.168	0.157
	Mung bean	RVI1	AGB = 0.054*RVI1+1.55	0.626	0.088	0.113
LAI	Red bean	RECI	LAI = 0.616*RECI+0.355	0.634	0.376	0.129
	Mung bean	B.450	LAI = –74.297*B.450+5.292	0.591	0.25	0.09

### Estimation Aboveground Biomass and Leaf Area Index Using Spectral Parameters Combined With Stepwise Multiple Linear Regression

To compare the growth parameter estimation models based on the optimal spectral parameters, we screened out 2–4 spectral parameters with a high correlation with the growth parameters of red bean and mung bean. Then, the SMLR method was used to establish AGB and LAI estimation models in the training set (Table 4). SMLR analysis showed that the models explained 85.7% (RMSE = 0.133 kg/m^2^, NRMSE = 0.125) and 75.7% (RMSE = 0.073 kg/m^2^, NRMSE = 0.093) of AGB variation in red bean and mung bean. Similar results were obtained for LAI. These models explained 69.8% (RMSE = 0.351, NRMSE = 0.121) and 67.2% of LAI (RMSE = 0.227, NRMSE = 0.081) variation in red bean and mung bean, respectively.

**TABLE 4:** Performance indicators of AGB and LAI estimation models established by the SMLR methods in the training set.

Growth parameters	Intercropping crops	Regression equation	Modeling accuracy		
			*R* ^2^	RMSE	NRMSE
AGB (kg/m^2^)	Red bean	AGB = 0.155*RVI1–27.913*B.450–0.964*MSR–5.09*G.555 + 2.748	0.857	0.133	0.125
	Mung bean	AGB = 0.231703*RVI1–1.1639*MSR–15.0778*B.450–3.64563*R.660 + 1.7216	0.757	0.073	0.093
LAI	Red bean	LAI = 0.478338*RECI–53.7192*B.450 + 0.123683*RVI1–1.12239*MSR+ 4.65337	0.698	0.351	0.121
	Mung bean	LAI = –49.2931*B.450–3.39808*SIPI/RVI1 + 4.98799	0.672	0.227	0.081

**FIGURE 5:**
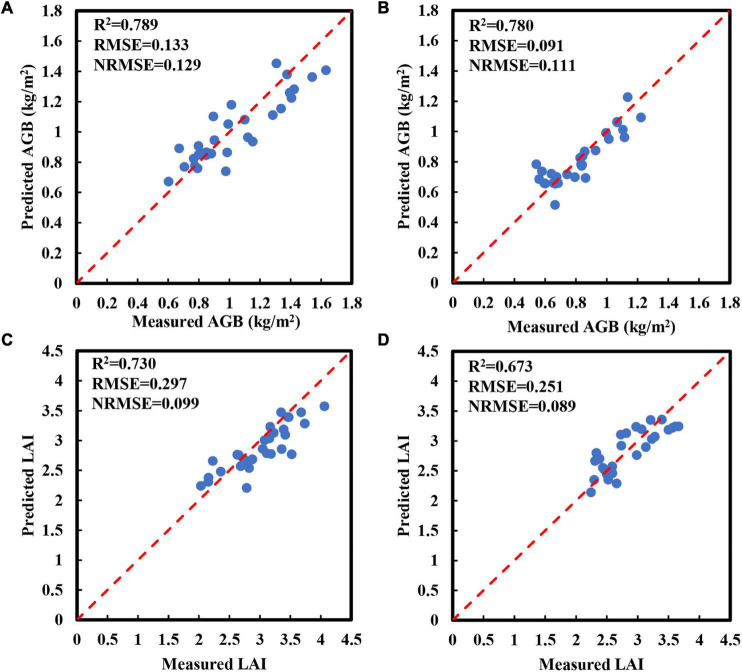
Relationship between the predicted and measured AGB and LAI obtained by using the SMLR models within the test dataset. **(A)** AGB of red bean; **(B)** AGB of mung bean; **(C)** LAI of red bean; **(D)** LAI of mung bean. The red line is a 1:1 line.

To evaluate the performance of the AGB and LAI estimation models constructed using SMLR, we plotted the relationship between the measured values and predicted values of AGB and LAI in the test dataset (Figure 5). Compared with the training set, the SMLR model showed a greater decrease in the explanatory degree of AGB variation, indicating that the estimation accuracy of the model decreased significantly. The NRMSE value increased to 0.129, indicating that the AGB estimation model of red bean was not stable. Compared with the training set, the SMLR model had lower explanatory power for AGB variation and higher NRMSE value, indicating that the accuracy of the estimation models of AGB of red bean decreased significantly and its stability was not good (Figure 5A). The accuracy of other models was basically consistent with the results of the training set, indicating that the stability of models was better. Compared with evaluation indexes of the LR models based on optimal spectral parameters, the *R*^2^ values of SMLR models based on multispectral parameters increased, while the RMSE and NRMSE values decreased. These results indicated that the performance of SMLR models was better than LR models in estimating the growth parameters of red bean and mung bean.

### Estimation of Aboveground Biomass and Leaf Area Index Using Spectral Parameters Combined With SVMs, Partial Least-Squares Regression, and Backpropagation Neural Network

**FIGURE 6:**
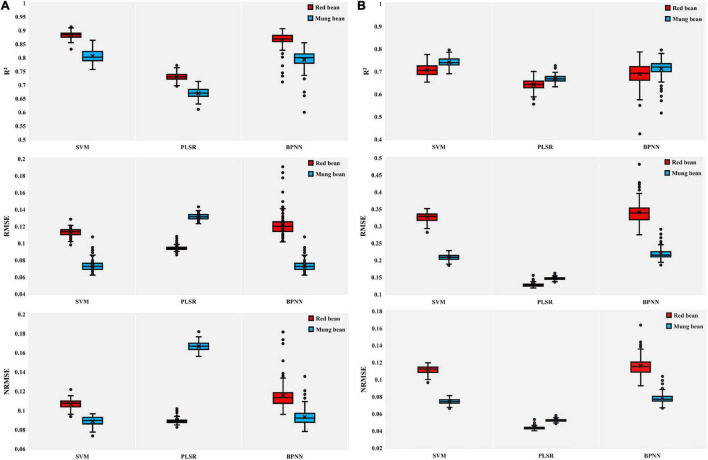
Boxplots for the coefficient of determination (*R*^2^), root-mean-square error (RMSE), and normalized RMSE (NRMSE) of the training results of SVM, PLSR, and BPNN models. **(A)** AGB of red bean and mung bean; **(B)** LAI of red bean and mung bean. The point plots indicate outliers encountered during the phase of the 100 different verifications repetitions and the black multiplication sign indicates the mean value.

**FIGURE 7:**
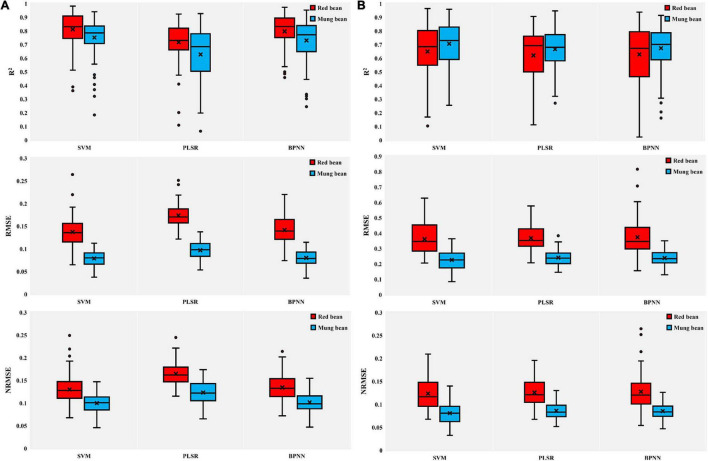
Box plots of coefficient of determination (*R*^2^), RMSE, and NRMSE of test results of SVM, PLSR, and BPNN. **(A)** AGB of red bean and mung bean; **(B)** LAI of red bean and mung bean. The point plots indicate outliers encountered during the phase of the 100 different test repetitions and the black multiplication sign indicates the mean value.

In addition, to evaluate the performance of SVMs, PLSR, and BPNN) in the estimation of crop growth parameters, we established AGB and LAI estimation models of red bean and mung bean by combining SVM, PLSR, and BPNN with spectral parameters. To prevent overfitting caused by using too many independent variables when establishing models, we selected five spectral parameters with high correlation for each growth parameter for modeling and analyzing according to the results of correlation analysis (Supplementary Figure 1). The training results given in Figure 6 indicated that the SVM method showed better performance than other methods in the estimation of the AGB and LAI of red bean and mung bean. Compared with PLSR and BPNN models, SVM models had the highest *R*^2^ values and relatively low RMSE and NRMSE values, indicating that SVM models had the highest accuracy in the estimation of the growth parameters of red bean and mung bean. Although BPNN also provided higher *R*^2^ values in the estimation of the growth parameters of red bean and mung bean, the obtained RMSE and NRMS values were higher with high variability. In addition, the accuracy of estimating the AGB of the red bean by three methods was better than that of mung bean, but the performance was the opposite in LAI estimation. The SVM models obtained the highest values of *R*^2^ and the lowest values of RMSE and NRMSE when estimating the growth parameters of red bean and mung bean in the test set (Figure 7). These results prove the excellent performance of the SVM models in estimating the growth parameters of red bean and mung bean. Similarly, the PLSR models were still the least applicable model for estimating the LAI and AGB of red bean and mung bean.

**FIGURE 8:**
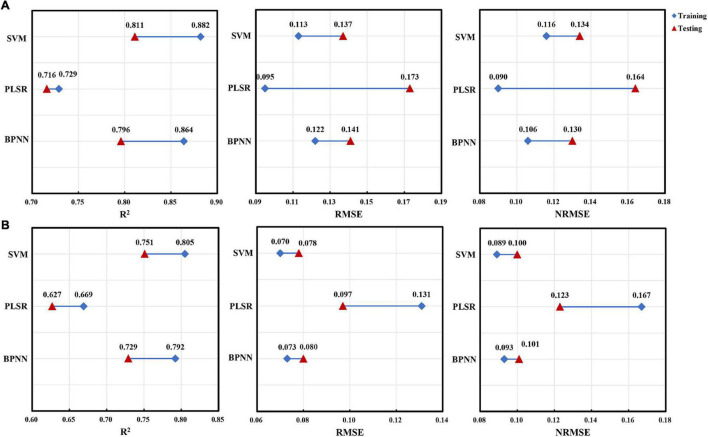
The difference between the performance indicators for AGB estimation of red bean and mung bean using three machine learning methods within training and test datasets. **(A)** Red bean; **(B)** mung bean.

**FIGURE 9:**
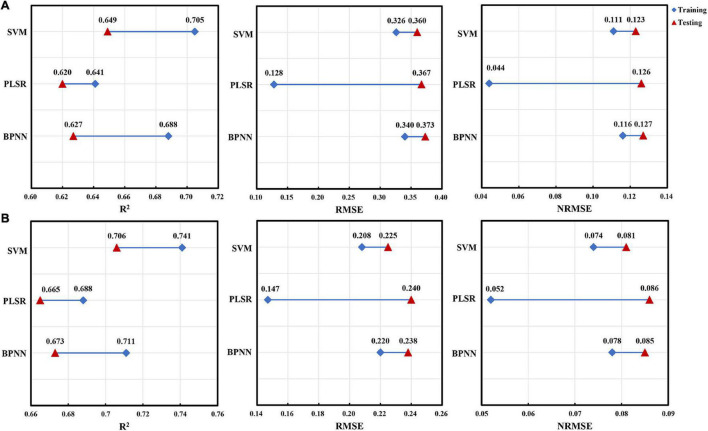
The difference between the performance indicators for LAI estimation of red bean and mung bean using three machine learning methods within training and test datasets. **(A)** Red bean; **(B)** mung bean.

To further compare the differences between the performance indicators calculated by using the training dataset and the test dataset in the three methods, we had drawn comparison charts of line segment connection (Figures 8, 9). For AGB of red bean, the SVM model showed high performance (Figure 8A). In the training set, the SVM model could explain 88.2% of the AGB variation in red bean, and the RMSE and NRMSE values were 0.113 and 0.116, respectively. In the test set, the explanatory degree of the SVM model for AGB variation decreased to 81.1%, RMSE and NRMSE increased to 0.137 and 0.134, respectively. Although the explanatory degree of AGB variation and RMSE value of the SVM models changed greatly, the prediction accuracy and stability of the models were better than that of PLSR and BPNN models. For AGB of mung bean, the SVM model showed better stability (Figure 8B). In both the training set and the test set, the SVM model had the highest explanatory degree (80.5 and 75.1%) of AGB variation and the lowest RMSE (0.070 and 0.078) and NRMSE (0.116 and 0.134). The difference between training results and test results was small, which is more stable than other models.

Similarly, the SVM model also showed high performance for LAI estimation of red bean and mung bean (Figure 9). In the training set and test set, the explanatory degrees of the SVM model for LAI variation were 70.5 and 64.9%, for RMSE were 0.326 and 0.360, and NRMSE were 0.116 and 0.134, respectively. The explanatory degrees of LAI for mung bean were 74.1 and 70.6%, RMSE were 0.208 and 0.225, NRMSE were 0.11 and 0.123, respectively. In terms of overall performance indicators, the SVM models had better accuracy than the PLSR model and BPNN model and had lower RMSE and NRMSE as well as small test differences.

## Discussion

### The Spectral Data Obtained From Unmanned Aerial Vehicle Multispectral Image Can Reliably Reflect the Growth Status of Crops Intercropped in Tea Plantation

Monitoring the growth of intercropping crops in tea plantations and guiding the formulation of tea plantation management measures using UAV-based multispectral imagery are very attractive. The results indicated that a single spectral parameter can be used to estimate the AGB and LAI of crops. However, the optimal spectral parameters for estimating growth parameters of red bean and mung bean were different, among which RVII could accurately estimate AGB, while RECI and B.450 were more suitable for estimating LAI. The difference between optimal spectral parameters for estimating AGB and LAI indicated that different VIs showed different sensitivities to AGB and LAI changes in different crops. Similarly, Li W. et al. (2020) found that RVI had a strong correlation with wheat biomass and LAI in the process of using meteorological factors and spectral information to study the disease measurement model of winter wheat, and Liu et al. (2019) also proved that RVI is an important VI for estimating biomass of winter oilseed rape. These conclusions were consistent with our results.

In addition, it was reported that LAI and AGB could exert a certain influence on the spectral reflectance of crop canopy in near infrared (NIR) and visible spectrum (Anthony et al., 2012; Liu et al., 2012; Jin et al., 2015). Qi et al. (2020) found that red and near-infrared bands were sensitive bands for LAI in the process of estimating the LAI of peanuts by using UVA multispectral images. According to the calculation formula of spectral parameters in Table 4, RVI1 is composed of red band and near-infrared band, and the red-edge chlorophyll index is composed of the red-edge band and near-infrared band. Jin et al. (2015) found that enhanced VI (EVI) with the blue band could estimate LAI and biomass more accurately than other spectral parameters when estimating LAI and biomass of wheat using multitemporal optical and radar parameters. In this study, the optimal spectral parameter B.450 used to estimate the LAI of mung bean represents the blue band, which is consistent with this result. In contrast, in the remote sensing monitoring of sorghum growth and development based on UAV system, Li et al. (2018) found that NDVI and RDVI showed a good exponential correlation with biomass; Shafian (2018) also proved that there was a high correlation between NDVI and LAI. Although the calculation of these two spectral parameters has a red band and near-infrared band, in our study, the correlation between these two spectral parameters and AGB and LAI of mung bean and red bean is not the highest, which may be due to some interference of shadow soil pixels in the process of extracting spectral parameters. Some studies also pointed out that the saturation problem of NDVI would reduce its function of predicting LAI under very high LAI values (Feng et al., 2020). However, the growth period of red bean and mung bean was relatively short and the growth rate was very fast, resulting in higher LAI data values collected later, which further leads to the low correlation between NDVI and the LAI of mung bean and red bean in this study.

### Different Machine Learning Algorithms Combined With Spectral Data Can Effectively Estimate the Growth Parameters of Intercropping Crops in Tea Plantation

In addition to single spectral parameters, SMLR, PLSR, SVM, and BPNN algorithms were used to monitor the growth parameters of intercropping crops in tea plantations. The results showed that the SMLR and PLSR models performed significantly better than the LR models, which is consistent with LAI estimation of peanut (Qi et al., 2020) and LAI and AGB estimation of winter wheat (Tao et al., 2020). The reason is that SMLR models and PLSR models use more spectral information related to the variables of interest than single spectral parameter models (Qin et al., 2017; Wei et al., 2018).

In addition, compared with the LR models based on a single spectral parameter or SMLR and PLSR models based on multiple parameters, the SVM and BPNN models can realize non-linear mapping between input and output variables. Therefore, the performance of the SVM and BPNN models in the estimation of growth parameters was better than other models. When the two models were compared, the SVM models still maintained excellent performance. Both the training results and test results of models maintained a high explanatory degree for AGB and LAI variations of red bean and mung bean. Because the SVM method is suitable for small samples, the BPNN method is usually used for a large number of sampled data (Zhu et al., 2019). However, the sample size used to construct models in this study is small, which highlights the superiority of the SVM method. In conclusion, the SVM model can effectively estimate the growth parameters of intercropping crops in tea plantations, and the fitting, stability, and accuracy of this model are better than other models. The superior performance of the SVM method observed in this study is consistent with previous results. For example, Yang X. et al. (2008) found that the SVM method had good learning ability and robustness in estimating the LAI of rice, while Yue et al. (2017) also proved that SVM had strong adaptability in estimating AGB of grassland. However, other studies have shown that PLSR provides better results than SVM in estimating crop growth parameters (Marabel and Alvarez-Taboada, 2013). This difference might depend on the degree of non-linearity in the relationships, the degree of multilinearity and noise in the independent variables, and how accurately the SVM parameters can be tuned (Christoffer et al., 2013). However, our crop growth data precisely fit the advantages of SVM in simulating non-linear relationships, thus highlighting the superiority of the SVM model in estimating growth parameters.

## Conclusion

Reasonable and reliable estimation of AGB and LAI is of great significance for monitoring crop growth and agricultural site-specific management decision-making. In this study, five machine learning algorithms (LR, SMLR, PLSR, SVM, and BPNN) were used to estimate AGB and LAI of red bean and mung bean in tea plantations based on the extracted multispectral image features collected by UAV remote sensing system. The results showed that the SVM and BPNN models, which can simulate non-linear relationships, were more accurate in estimating AGB and LAI of red bean and mung bean compared with simple LR, SMLR, and PLSR models. In particular, the SVM model provides higher performance in the estimation of AGB and LAI of red bean and mung bean. Both RMSE and NRMSE of the training set and test set were smaller, and the explanatory degree for AGB and LAI variation was higher. It is proved that the use of UAV multispectral image data combined with machine learning methods can effectively monitor the growth status of crops in tea plantations and provide valuable practical guidelines for site-specific tea plantations and the improvement of their ecological and environmental benefits.

## Data Availability Statement

The original contributions presented in the study are included in the article/Supplementary Material, further inquiries can be directed to the corresponding author/s.

## Author Contributions

YS carried out the experiment, collected and processed the data, and wrote the manuscript. ZD and KF raised the hypothesis underlying this study, designed the experiment, helped organize the manuscript structure, and directed the study. YG and YW participated in designing the experiment and reviewed the manuscript. SC and DL participated in designing the experiment and directed the study. All authors contributed to the study and approved the submitted version.

## Conflict of Interest

The authors declare that the research was conducted in the absence of any commercial or financial relationships that could be construed as a potential conflict of interest.

## Publisher’s Note

All claims expressed in this article are solely those of the authors and do not necessarily represent those of their affiliated organizations, or those of the publisher, the editors and the reviewers. Any product that may be evaluated in this article, or claim that may be made by its manufacturer, is not guaranteed or endorsed by the publisher.
