# Supplementary material for: Using Unmanned Aerial Vehicle-Based Multispectral Image Data to Monitor the Growth of Intercropping Crops in Tea Plantation
Source: Front Plant Sci. 2022 Feb 25;13:820585. doi: 10.3389/fpls.2022.820585 (PMC8914207; doi:10.3389/fpls.2022.820585)

Supplementary Material

# Supplementary Figures and Tables

## Supplementary Figures

**Supplementary Figure 1.** Correlation between growth parameters and spectral parameters selected for modeling. (A). Correlation between spectral parameters and AGB of red bean; (B). Correlation between spectral parameters and LAI of red bean; (C). Correlation between spectral parameters and AGB of mung bean; (D). Correlation between spectral parameters and LAI of mung bean.
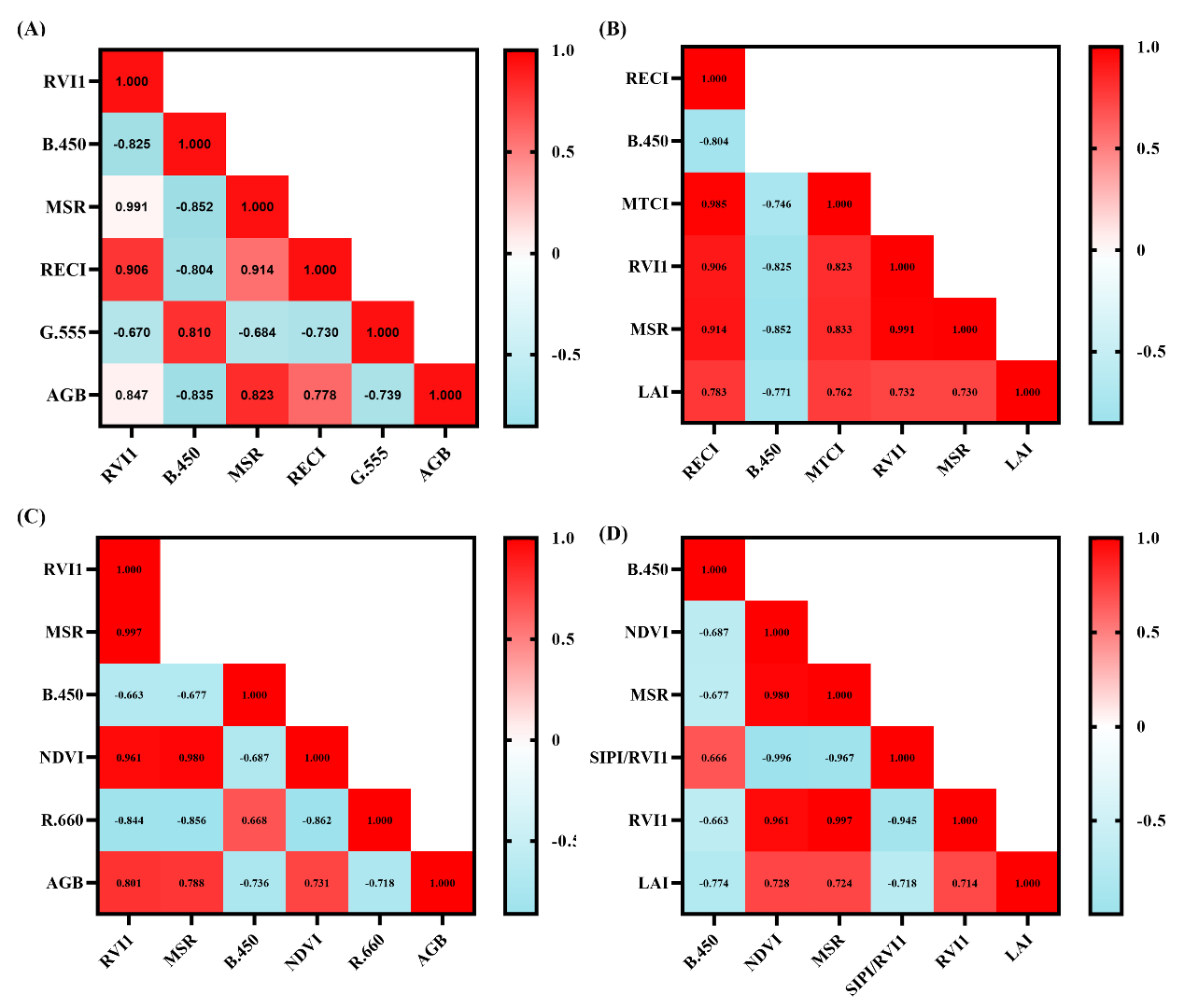

Supplement: Supplementary file 1 [file Table_1.DOCX]
